# Near-infrared and mid-infrared semiconductor broadband light emitters

**DOI:** 10.1038/lsa.2017.170

**Published:** 2018-03-23

**Authors:** Chun-Cai Hou, Hong-Mei Chen, Jin-Chuan Zhang, Ning Zhuo, Yuan-Qing Huang, Richard A Hogg, David TD Childs, Ji-Qiang Ning, Zhan-Guo Wang, Feng-Qi Liu, Zi-Yang Zhang

**Affiliations:** 1 Key Lab of Nanodevices and Applications, Suzhou Institute of Nano-Tech and Nano-Bionics, Chinese Academy of Sciences, Suzhou 215123, China; 2 Key Laboratory of Semiconductor Materials Science, Institute of Semiconductors, Chinese Academy of Sciences, Beijing 100083, China; 3 College of Materials Science and Opto-Electronic Technology, University of Chinese Academy of Sciences, Beijing 100049, China; 4 School of Engineering, The University of Glasgow, Glasgow G12 8LT, UK; 5 Vacuum Interconnected Nanotech Workstation, Suzhou Institute of Nano-Tech and Nano-Bionics, Chinese Academy of Sciences, Suzhou 215123, China

**Keywords:** broadband light emitters, optical coherence tomography, quantum cascade structure, quantum dot

## Abstract

Semiconductor broadband light emitters have emerged as ideal and vital light sources for a range of biomedical sensing/imaging applications, especially for optical coherence tomography systems. Although near-infrared broadband light emitters have found increasingly wide utilization in these imaging applications, the requirement to simultaneously achieve both a high spectral bandwidth and output power is still challenging for such devices. Owing to the relatively weak amplified spontaneous emission, as a consequence of the very short non-radiative carrier lifetime of the inter-subband transitions in quantum cascade structures, it is even more challenging to obtain desirable mid-infrared broadband light emitters. There have been great efforts in the past 20 years to pursue high-efficiency broadband optical gain and very low reflectivity in waveguide structures, which are two key factors determining the performance of broadband light emitters. Here we describe the realization of a high continuous wave light power of >20 mW and broadband width of >130 nm with near-infrared broadband light emitters and the first mid-infrared broadband light emitters operating under continuous wave mode at room temperature by employing a modulation p-doped InGaAs/GaAs quantum dot active region with a ‘J’-shape ridge waveguide structure and a quantum cascade active region with a dual-end analogous monolithic integrated tapered waveguide structure, respectively. This work is of great importance to improve the performance of existing near-infrared optical coherence tomography systems and describes a major advance toward reliable and cost-effective mid-infrared imaging and sensing systems, which do not presently exist due to the lack of appropriate low-coherence mid-infrared semiconductor broadband light sources.

## Introduction

Semiconductor broadband light emitters (BLEs) are edge-emitting light sources that utilize the internal amplification of spontaneous emission (ASE). The unique property of BLEs is the combination of the high brightness of a laser diode with a broad optical spectrum and hence the low coherence of a light-emitting diode. BLEs have emerged as the ideal core optical sources for numerous industrial and medical applications, such as fiber-optic gyroscopes and sensors, wavelength-division multiplexing system testing, and especially optical coherence tomography (OCT) systems^1, 2, 3, 4^.

Recently, such broadband light sources operating in the near-infrared (NIR) region have been successfully employed in OCT for clinical ophthalmology and skin disease diagnosis^5, 6^. However, there is always a trade-off between the high-power output and wide spectral bandwidth of the BLE devices. Therefore, it is very challenging to broaden the emission bandwidth (full width at half maximum (FWHM)) beyond ~100 nm while keeping a high continuous wave (CW) power output of >10 mW^7, 8^. Thus, currently, the main challenge for the development of NIR-OCT imaging techniques is to simultaneously widen the bandwidth and increase the light power of the light sources and hence enhance the axial resolution of the images and the penetration depth in tissues^9, 10^.

Compounds such as collagen amide, phosphate and carbonate absorb relatively little within the spectrum covered by existing NIR-OCT systems, and therefore the spectral response to molecular species in the NIR region comprises very weak overtones and a combination vibrational absorption bands. In contrast, the mid-infrared (MIR) spectral region is dominated by fundamental absorption bands specifically attributable to these compounds. Therefore, the characteristic MIR absorption by these biochemical species provides a potentially powerful, sensitive and specific method of imaging tissue, such as bone, tendon and stratum corneum of the skin^11, 12, 13^. Presently, broadband MIR light sources rely on the generation of light by a high-power laser pumped by chalcogenide or fluoride fibers^14, 15, 16^. However, such devices are bulky, expensive and complex, which severely impede their widespread development. As a consequence, the lack of an appropriate MIR-BLE has inhibited the development of MIR-OCT systems^17^. Semiconductor BLEs are compact, robust and easy to operate, lending themselves to low-cost mass production, and therefore have great potential to realize such a MIR-OCT imaging system.

In this paper, two new developments in NIR- and MIR-BLEs are reported. Multi-stacked modulation p-doped quantum dot (QD) layered structures and quantum cascade (QC) structures are employed to fabricate the NIR-BLE and MIR-BLE, respectively. An NIR-QD-BLE with a high CW power (>20 mW) and wide bandwidth (FWHM>130 nm) is demonstrated. This simultaneous record high power and broad bandwidth is mainly attributed to the enhanced broadband optical gain by introducing modulation p-doping to the QD structures. However, in contrast to the NIR-BLE based on interband transitions, the radiative efficiency of spontaneous emission is very low (~10^−3^) in intersubband transitions owing to the long spontaneous lifetime (*τ*_SR_~μs–ns) compared with the very short non-radiative lifetime (*τ*_NR_~ps). As spontaneous emission is significantly reduced in MIR-BLEs compared with NIR-BLEs, the reflectivity, which is generally required for a radiative efficiency ~<10^−3^ for the NIR-BLEs^18, 19, 20^, obviously cannot meet the requirement for MIR-BLEs. Therefore, compared with NIR-BLEs, in order to achieve high power, the exponential function needs to be significant in MIR-BLEs. The device mirror loss determines the onset of parasitic lasing, and therefore long devices or high modal gain places a strong emphasis on the facet reflectivity.

As mentioned above, obtaining an ultralow reflectivity (*R*) is crucial. To obtain the demanding *R*, in this work, a focused ion beam (FIB) milling technique was utilized to form different facet angles on one cleaved facet in the above ‘J’-shape MIR-QC devices. By combining the length-dependent characterization of QC laser materials, the obtained *R* was calculated to be ~<10^−6^. Such low *R* normally requires complicated waveguide structures combining two or more methods to suppress device lasing, such as introducing a rounded sloped wet etched facet and tilted waveguide structures or an antireflection coating and spiral cavity structures with large bend radius^21^. These approaches are always at the expense of light emission from one of the two emitting facets, resulting in substantial optical loss, a serious heating effect, low conversion efficiency and less diversity of the device structures. Until now, all previous QC-BLE devices operated only under pulsed mode operation with very low duty cycle^17, 21, 22^, and among them, only one device has been shown to function at room temperature (RT)^21^. In this work, a monolithic integrated waveguide structure with the tilted BLE and an optical amplifier is designed and fabricated, leading to the first working RT-CW MIR-QC-BLE. In addition, simultaneous dual broadband light emission with different power-, different optical bandwidth- and different temperature-dependent characteristics are observed from the two emitting facets in the device.

## Materials and methods

**Figure 1:**
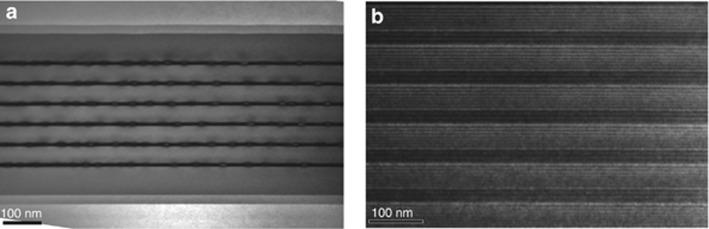
Cross-sectional transmission electron microscopic images of (**a**) the multiple InAs/GaAs quantum dot active layer structure and (**b**) the In_0.678_Ga_0.322_As/In_0.365_Al_0.635_As quantum well cascade active layer structure.

The QD and QC device structures were grown in a solid-source molecular beam epitaxy system on GaAs and InP substrates, respectively. Two kinds of six-layer InAs/GaAs (un-doped and p-doped) QD structures were fabricated. For the un-doped QD laser structure, each QD layer consisted of 2.7 monolayers of InAs covered with 6 nm of In_0.15_Ga_0.85_As strain reducing layer. The six InAs/InGaAs QD layers were separated by 50-nm GaAs barriers, as seen in Figure 1a. The entire QD structure was sandwiched by 1500-nm n-Al_0.4_Ga_0.6_As and 1500-nm p-Al_0.4_Ga_0.6_As cladding layers. The p-doped QD sample was grown with an identical structure, except modulation p-doping with Be at a concentration of 30 acceptors per dot was located in a 6-nm wide GaAs layer 10-nm beneath each QD layer. The surface density of the QD used here was ~4 × 10^10^ cm^−2^. The QC structure was grown in a single growth step on an InP substrate using a strain-compensated In_0.678_Ga_0.322_As/In_0.365_Al_0.635_As material based on a two-phonon resonance design. The entire structure is as follows: 1.2-μm lower cladding (Si, 2.2 × 10^16^ cm^−3^), 0.3-μm thick n-In_0.53_Ga_0.47_As layer (Si, 8 × 10^16^ cm^−3^), 30 stages of strain-compensated In_0.678_Ga_0.322_As/In_0.365_Al_0.635_As quantum wells and barriers, 0.3-μm thick n-In_0.53_Ga_0.47_As layer (Si, 8 × 10^16^ cm^−3^), 2.4-μm upper cladding (Si, 2.2 × 10^16^ cm^−3^), and 0.6-μm cap cladding (Si, 1 × 10^19^ cm^−3^). Thirty repetitions of the injector/active region were sandwiched by low-doped (*n*≈8 × 10^16^ cm^−3^) InGaAs layers, as seen in Figure 1b. The layer sequence of each period, starting from the injection barrier, is as follows (thickness in nanometers): **3.8**/1.2/**1.3**/4.3/**1.3**/3.8/**1.4**/3.6/**2.2**/2.8/**1.7**/2.5/**1.8**/2.2/**1.9**/2.1/**2.1**/2.0/**2.1**/1.8/**2.7**/1.8. In_0.365_Al_0.635_As barrier layers are in bold, In_0.678_Ga_0.322_As quantum well layers are in regular type and doped layers (Si, 1.5 × 10^17^ cm^−3^) are underlined.

The QD samples were processed into 5-μm wide ‘J’-shape ridge waveguide structures by a dry etching process, and the QC samples were processed into 10-μm wide ‘J’-shape ridge waveguide structure and a monolithic integrated ridge waveguide structure with a 0.6-mm long 17° tilted BLE and a 2.4-mm long 17° tilted optical amplifier with a flare angle of 2° by a one-step wet etching process. Ti/Au and Au/Ge/Ni/Au provided the top and bottom Ohmic contacts, respectively, for both the QD- and QC-BLEs. The BLE chips were mounted on indium-plated copper tiles without antireflection coating on the facets. All the ‘J’-shape ridge waveguide QD and QC devices were positioned epitaxial-side up, while the analogous monolithic integrated ridge waveguide QC devices were positioned epitaxial-side down.

## Results and discussion

**Figure 2:**
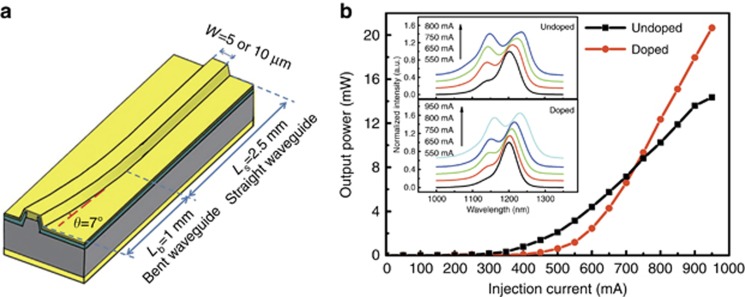
(**a**) Schematic device diagrams of the ‘J’-shaped QD and QC devices. (**b**) P–I characteristics of the un-doped and p-doped QD-BLEs measured at RT under CW operation. Insets: the corresponding emission spectra of the QD devices under various injection currents.

Figure 2a shows schematic diagrams of the ‘J’-shape ridge waveguide QD and QC device structures, in which the light propagating toward the rear end of the device is reflected and undergoes double-pass amplification. Therefore, it is usually utilized to pursue high-power output in BLEs. As seen in Figure 2a, the bending region has a radius of curvature of 0.8 cm to minimize optical propagation loss and is tilted from the normal direction of the cleaved facet by 7° to reduce the reflection and suppress lasing. As long devices are more suitable for achieving higher power, 3.5-mm long cavities were chosen, consisting of a 1-mm bending region and 2.5-mm straight region. For the NIR-QD devices, the 5-μm wide J-shape ridge waveguide structure (beneficial to fiber coupling) was fabricated by inductively coupled plasma dry etching. Considering the high confinement effect of the optical mode and low damage to the active region by dry etching, the ridge waveguide was etched to ~200 nm above the QD active region. In contrast, for the MIR-QC devices, owing to the pronounced current spreading by the strong discontinuities of the anisotropic electrical conductivity of the multi-quantum well active region^23^, the QC ‘J’-shape ridge waveguide was sufficiently etched ~7 μm by wet chemical etching to expose the active core. The width of the ‘J’-shape ridge waveguide QC-BLE is 10 μm.

The light–current characteristics of the un-doped and p-doped QD devices were measured under CW operation at RT, as seen in Figure 2b. The insets show the corresponding electroluminescence spectra at various injection currents for the two samples. At low injection current, the ground state (GS) of the QDs centered at ~1200 nm dominates the emission spectrum, with a spectra bandwidth of a few tens of nanometers. With an increasing injection current, the GS gradually approaches saturation, and the excited state (ES) emission appears. For the un-doped sample, at a current of 800 mA, a 141-nm wide spectrum is obtained from a simultaneous contribution of the GS and ES, while a larger current is needed for the p-doped QD-BLE to achieve the GS=ES regime with a 134-nm bandwidth. A higher maximum light power of 21 mW and higher slope efficiency of 0.056 W A^−1^ in the p-doped QD device compared with 14 mW and 0.03 W A^−1^, respectively, in the un-doped QD device are observed in Figure 2b. These improvements are mainly due to the enhanced modal gain in the QDs by the increasing hole population in the valence band by the introduction of tens of acceptors per QD^24^. However, the p-doping usually tends to enhance monomolecular (through dopant-related defects), radiative and Auger (through increased hole population) recombination^25, 26^, and among them, Auger recombination is the main non-radiative mechanism in QDs. Thus the turn-on current was found to increase from ~375 mA for the un-doped QD-BLE to ~500 mA for the p-doped structure. Currently, although NIR-BLEs have found increasingly wide utilization in OCT imaging applications, the simultaneous demand for a wide bandwidth and high output power is still challenging for such devices. Here a high CW light power of >20 mW with an FWHM >130 nm was synchronously obtained in the narrow ridge waveguide p-doped QD-BLEs, which will significantly simplify the design and reduce the cost of current NIR-OCT systems.

**Figure 3:**
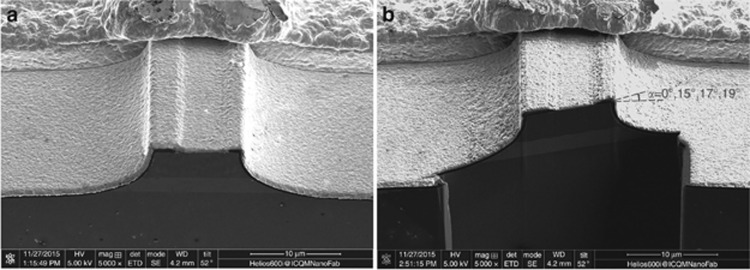
Scanning electron microscope (SEM) images of the MIR devices before (**a**) and after (**b**) focus ion beam milling.

The ‘J’-shape ridge waveguide MIR-QC devices were characterized under pulsed mode operation (1-μs pulsed width at a repetition rate of 5 kHz). However, in contrast to the previously discussed QD-BLEs, the MIR-QC devices exhibited lasing characteristics from 80 to 300 K, indicating that the ‘J’-shape structure could not provide a sufficient low reflectivity to suppress lasing to allow broadband superluminescent light output. As the Brewster angle is calculated to be ~17.4° for the MIR-QC devices, in order to further reduce the reflectivity, an FIB was used to mill different angles of 15°, 17° and 19° in the straight part, as seen in Figure 3b. The FIB milling was performed with a Helios NanoLab 600 i FIB/scanning electron microscopy system using a probe current of ~790 pA, a step size of 38.5 nm and a dwell time of 1 μs. The facets were milled from the cleaved plane to a >15 μm depth to inhibit light reflect back from the bottom surface into cavity.

**Figure 4:**
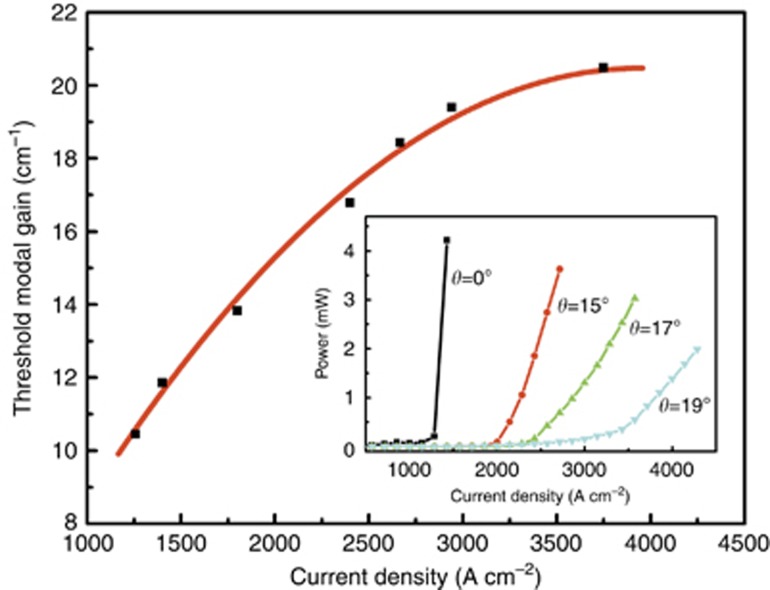
Threshold modal gain of the ‘J’-shaped QC lasers as a function of the current density. Inset: the light–injection current density characteristics of the MIR-QC devices with different rear facet angles of 0°, 15°, 17° and 19°.

The inset of Figure 4 shows the light–injection current density characteristics of the MIR-QC devices with different rear facet angles of 0°, 15°, 17° and 19°. All the measurements were performed at 80 K under pulsed operation (1-μs pulsed width and 5 kHz repetition rate). The threshold current density (*J*_th_) of the device before the FIB milling is 1.3 kA cm^−2^, and the lasing position is ~4 μm at 80 K. After the FIB milling, the devices with rear facet angles of 15° and 17° still exhibited lasing characteristics with increased *J*_th_ (2 and 2.4 kA cm^−2^) owing to the reduced effective facet reflectivity from the angled facets. By further increasing the etched angle to 19°, a ~50-cm^−1^ broadband emission spectrum was obtained. Usually, the effective facet reflectivity of the facet of the light-emitting device is to be determined by measuring the Fabry–Perot modulation depth. However, in our test system, it is very difficult to adopt this measurement to calculate the reflectivity as the separation is smaller than the resolution. Therefore, we utilized a new method to experimentally obtain the effective facet reflectivity by plotting the curve of current density versus gain of QC material, which is similar to that previously utilized in NIR-QD devices^27^. The ‘J’-shape MIR-QC lasers with cavity lengths of 1.6, 1.7, 1.8, 2, 2.5, 3 and 3.5 mm were selected to study the length-dependent characteristics of the threshold current density. By using the standard laser threshold equation, 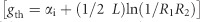, the QC modal gain as a function of the injection current density was obtained, as shown in Figure 4. The coefficient of internal optical loss, *α*_i_, of this MIR-QC laser material is determined to be ~2 cm^−1^. Assuming the effective reflectivity *R*_1_ of the front facet is ~1%^28^, using the above equation, the observed lasing threshold current density of the MIR-QC devices in the inset of Figure 4 allow the determination of the effective reflectivity *R*_2_ of the FIB etched angled facets. Reflectivities of 8.4 × 10^−3^ and 3.0 × 10^−3^ are calculated for facets with angles of 15° and 17°, respectively, and a lower reflectivity of 3.1 × 10^−4^ is achieved in the device with a 19° angled facet. We note that the gain of the QC active region saturates at a value of ~20 cm^−1^. To achieve the highest possible power, we aim to operate as close to this value as possible, which would require a combined effective facet reflectivity of <10^−6^ from the two facets.

**Figure 5:**
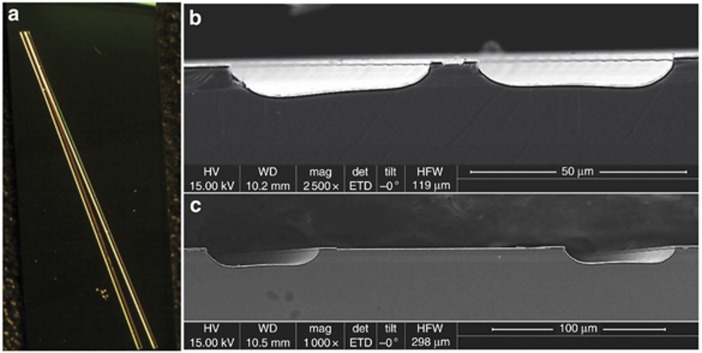
(**a**) Top-view microscopic image of the device. SEM images of the narrow facet (**b**) and the wide facet (**c**) of the broadband QC device.

We designed a new monolithic integrated waveguide device structure with a tilted BLE and an optical amplifier to ensure low reflectivity of <10^−6^ and light emission from both the dual ends. Considering that the longer straight waveguide may be deleterious to suppress lasing and that the longer amplifier part has enhanced optical amplification, the waveguide device structure chosen here consists of a short straight section with a length of 0.6 mm followed by a large tapered section with a length of 2.4 mm. Figure 5a shows a top-view microscopic image of the fabricated broadband QC device, in which the total 3-mm long waveguide is tilted by 17° with respect to the normal facet. The scanning electron microscopic images of the two emitting facets are shown in Figure 5b and 5c, indicating that the width of the narrow facet is ~9 μm and that the width of wide end is ~120 μm, respectively. To further relieve the problem of self-heating, the devices with a double-trench waveguide structure were mounted epitaxial-side down on a heat sink.

**Figure 6:**
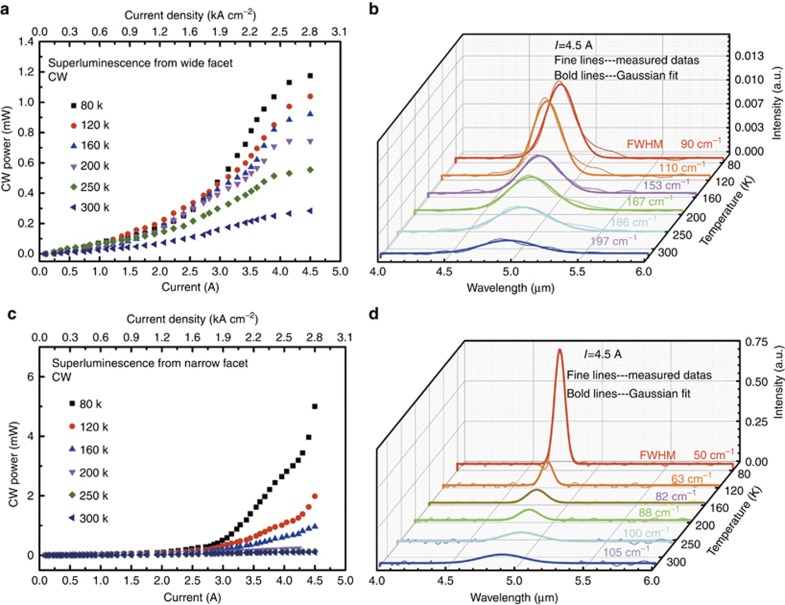
(**a**) and (**c**) Light–current characteristics of the wide and narrow emitting facets and (**b**) and (**d**) the corresponding emission spectra from the wide and narrow emitting facets, respectively, measured under CW operation mode at different temperatures from 80 to 300 K (*I*=4.5 A).

Figure 6 shows the light–current characteristics and emission spectra measured at different temperature from 80 to 300 K of the monolithic integrated QC waveguide device, where the output power and spectra were collected from both the narrow and wide facets. Lasing was completely suppressed, and broadband light emission was observed from the dual-emitting facets, in agreement with the simulation results and the results of the FIB-milled angled facet devices. Therefore, the basic requirement of reflectivity of 10^−6^ to achieve the broadband QC devices is further confirmed.

In contrast to previous reports (most the measurements were performed under pulsed mode operation with low duty cycle of ~0.05%^17, 21^), to the best of our knowledge, this is the first time that the measurements are performed in CW mode, which is critical for practical applications. In addition, the optical emission from our devices in the Dewar was directly collimated by an HgCdTe detector with a corrected collection efficiency of 10%.

The fabrication and potential applications for MIR broadband QC light emitters were proposed by Gmach *et al*^29^ in 2002. Over the past 15 years, great effort has been made to improve the device performance to pursue RT-CW working devices. However, as there exist significant challenges for suppressing lasing and obtaining broadband light emissions mentioned above, most approaches have always been at the expense of light emission from one of the two emitting facets, resulting in large optical loss, serious heating effects and low conversion efficiency. Therefore, the working currents (turn-on current of ~4.5 A (4 kA cm^−2^)) have been high, making RT-CW operation unattainable^21^.

For our QC devices, first, broadband light emission behavior from both the wide and narrow facets is evidenced by a superlinear increase in optical power with an increasing current and their corresponding emission spectra. Very low turn-on currents of 0.5 A (0.3 kA cm^−2^) and 1.5 A (0.9 kA cm^−2^) are obtained from the wide and narrow facets, respectively. In comparison to previously reported broadband QC devices, the low turn-on current results from a lower optical loss, leading to low self-heating effects. Maximum powers of 5 and 1.2 mW were obtained from the narrow and wide facets, respectively, at 80 K. By further increasing the injection current, roll-over starts to appear, as seen in Figure 6a and 6c, and the device does not exhibit lasing for all currents used in the measurements.

Interestingly, both the light-power and emission spectral characteristics are observed to be quite different from the narrow and wide emitting facets of the QC device. As shown in Figure 6a and 6c, the light power from the narrow facet is much higher than that from the wide facet at low temperature, while, as seen in Figure 6b and 6d, the spectrum from the narrow facet is also much narrower than that from the wide facet. For the monolithic integrated QC waveguide device, the reflectivity of the narrow facet is ~10^−2^ and that of the wide facet is ~10^−4^. The reflectance of the narrow facet is not negligible, and light reflected from the narrow facet will experience double-pass gain and exit from the wide facet. For the narrow facet, optical wave propagating from the tapered section to the straight section provides a high level of ‘seeds’ to produce a high level of superluminescent light emission. Notably, there exists reflectivity at the interface of the tilted straight and tapered sections. The tapered waveguide is a linearly tapered stripe, and thus there may be a modulation of the modal index. Therefore, the straight waveguide could play the role of a resonant cavity. In such a cavity, only the photons satisfying the Fabry–Perot conditions are significantly amplified and are subsequently emitted from the narrow facet. In this process, the amplification level of the spontaneous emission is much higher than that of the double-pass gain. The experimental result that the turn-on current of the wide facet emitting (0.5 A) is smaller than that of the narrow facet emitting (1.5 A) verifies the above two differential amplification processes. However, the lasing point is not reached, as the low reflectivity could not offer sufficient feedback. Therefore, compared with the spectrum from wide facet, the spectrum from the narrow facet features higher power but a smaller FWHM. In addition, the output power and slope efficiency from the narrow facet are obviously decreased from 80 to 300 K. This is because the heat accumulation can lead to an increase of the refractive index in the tapered section with a larger area. Therefore, the amount of optical wave propagating from the tapered section to the straight section will be reduced, resulting in poor temperature stability compared with that from the wide facet.

Figure 6b and 6d, shows the emission spectra at different temperatures taken with a Fourier transform infrared spectrometer in step-scan mode with a resolution of 8 cm^−1^. The FWHMs of the Gaussian-shaped spectra are ~50 cm^−1^ at 80 K and ~105 cm^−1^ at 300 K from the narrow facet, and the FWHMs from the wide facet are ~90 cm^−1^ at 80 K and ~197 cm^−1^ at 300 K. All the spectra exhibit a flat center region, indicating their suitability as broadband ASE sources. For biomedical imaging applications in OCT, in addition to the optical power, the coherence length of the source plays a critical role because a smaller coherence length results in a higher axial resolution of the 3D images.

**Figure 7:**
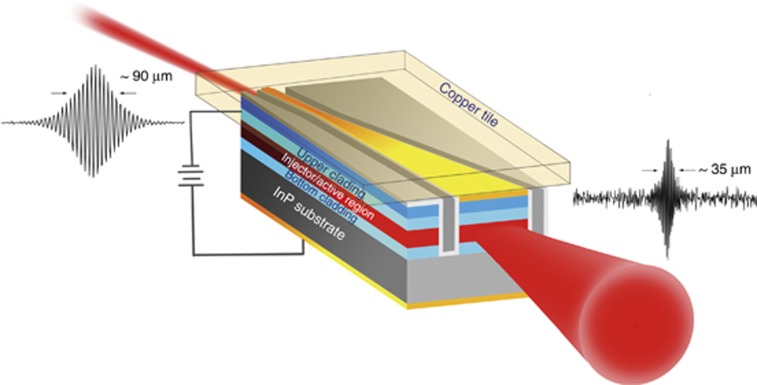
Schematic diagram of the two simultaneous incoherent light emissions in the MIR-QC broadband light emitter.

Figure 7 shows a schematic figure for the QC broadband light emitter, in which two different types of broadband light emit from the two facets. From the interferograms of the device emission, the coherence lengths from the wide facet are determined to be ~110 and ~35 μm at 80 and 300 K, respectively, and the coherence lengths from the narrow facet are ~200 and ~90 μm at 80 and 300 K, respectively. The results indicate that the dual-end emitting device structure not only provides a major advance toward high-performance MIR broadband light sources but also enriches the diversity of the working devices. As the broadband emission spectra are Gaussian in shape from both the narrow and wide facets, they exhibit good suitability as broadband ASE sources for OCT imaging application. For example, the wide-facet emission could be used for free-space systems, and the narrow emission could be used for fiber-based systems.

## Conclusions

In this work, a high-power and broadband p-doped NIR-QD-BLE was developed using a ‘J’-shape ridge waveguide device structure. By further minimizing the reflectivity to <10^−6^ using an analogous monolithic integrated waveguide structure with a tilted BLE and an optical amplifier, the first CW MIR-QC-BLE at RT was demonstrated. For the MIR-BLE, two different low-coherence sources were simultaneously obtained from the dual-emitting facets. High-performance semiconductor BLEs in the NIR and MIR ranges are the core and vital optical sources in OCT systems. These results will simplify the design and significantly reduce the cost of current NIR-OCT systems and provide a new opportunity toward achieving MIR-OCT systems, which do not presently exist due to the lack of appropriate MIR semiconductor broadband light sources.
